# Coding undiagnosed rare disease patients in health information systems: recommendations from the RD-CODE project

**DOI:** 10.1186/s13023-024-03030-2

**Published:** 2024-01-27

**Authors:** Céline Angin, Monica Mazzucato, Stefanie Weber, Kurt Kirch, Waed Abdel Khalek, Houda Ali, Sylvie Maiella, Annie Olry, Anne-Sophie Jannot, Ana Rath

**Affiliations:** 1 French National Rare Disease Registry (BNDMR), Greater Paris University Hospitals (AP-HP), 33 Boulevard de Picpus, 75012 Paris, France; 2 RD Coordinating Centre, Veneto Region, Padua University Hospital, Padua, Italy; 3 BfArM, Cologne, Germany; 4 Inserm, US14-Orphanet Paris, France; 5 Centre de Recherche Des Cordeliers Paris, Université Paris Cité, HeKA INSERM, INRIA Paris, Paris, France

**Keywords:** Rare diseases, Undiagnosed patients, Coding, ORPHAcodes, Health information systems, Diagnosis, Electronic health records, Registries, Public health, Statistics, Epidemiology

## Abstract

**Background:**

In European Union countries, any disease affecting less than 5 people in 10,000 is considered rare. As expertise is scarce and rare diseases (RD) are complex, RD patients can remain undiagnosed for many years. The period of searching for a diagnosis, called diagnostic delay, sometimes leads to a diagnostic dead end when the patient’s disease is impossible to diagnose after undergoing all available investigations. In recent years, extensive efforts have been made to support the implementation of ORPHA nomenclature in health information systems (HIS) so as to allow RD coding. Until recently, the nomenclature only encompassed codes for specific RD. Persons suffering from a suspected RD who could not be diagnosed even after full investigation, could not be coded with ORPHAcodes. The recognition of the RD status is necessary for patients, even if they do not have a precise diagnosis. It can facilitate reimbursement of care, be socially and psychologically empowering, and grant them access to scientific advances.

**Results:**

The RD-CODE project aimed at making those patients identifiable in HIS in order to produce crucial epidemiological data. Undiagnosed patients were defined as patients for whom no clinically-known disorder could be confirmed by an expert center after all reasonable efforts to obtain a diagnosis according to the state-of-the-art and diagnostic capabilities available. Three recommendations for the coding of undiagnosed RD patients were produced by a multi-stakeholder panel of experts: 1/ Capture the diagnostic ascertainment for all rare disease cases; 2/ Use the newly created ORPHAcode (ORPHA:616874 “Rare disorder without a determined diagnosis after full investigation”), available in the Orphanet nomenclature: as the code is new, guidelines are essential to ensure its correct and homogeneous use for undiagnosed patients’ identification in Europe and beyond; 3/ Use additional descriptors in registries.

**Conclusions:**

The recommendations can now be implemented in HIS (electronic health records and/or registries) and could be a game-changer for patients, clinicians and researchers in the field, enabling assessment of the RD population, including undiagnosed patients, adaptation of policy measures including financing for care and research programs, and to improved access of undiagnosed patients to research programs.

## Background

In European Union (EU) countries, any disease affecting no more than 5 people in 10,000 is considered rare [1]. It is estimated that the 6000 to 7000 distinct rare diseases (RD) affect up to 5.9 per cent of the general population, corresponding to an estimated 300 million people worldwide [2]. This estimation is based on epidemiological data curated from the literature and registries in the Orphanet database [3]. However, rare disease population identification remains a challenge. The main hospital code systems such as ICD [4] (the International Statistical Classification of Diseases and Related Health Problems maintained and published by the World Health Organisation) or SNOMED CT [5] (Systematized Nomenclature of Medicine Clinical Terms) lack specific codes to code rare disorders: only 8% of rare diseases have a specific ICD-10 code (the 10th version of ICD) and there is no “rare” flag in SNOMED CT or ICD nor a dedicated classification chapter or block. As a consequence, rare diseases are currently under-reported, under-recognized and thus under-resourced in health care systems.

Orphanet has developed and maintains a rare disease-specific nomenclature, the ORPHA nomenclature, organized in a multi-hierarchical classification that allows a precise representation of rare diseases. Each clinical entity is assigned a unique and stable identifier over time, the ORPHAcode. This nomenclature is released annually together with release notes and tools in a comprehensive “Nomenclature Pack" [6]. Although the adoption of the Orphanet nomenclature in a growing number of Hospital Information Systems through Europe and beyond is a great step forward to give visibility to rare disease patients [7–11], it did not allow the identification of undiagnosed rare disease patients until the implementation of the recommendations we present in this article.

The time to reach a diagnosis is an interesting public health indicator, as it may have serious unintended consequences for both mental and physical health of the patients and their families and negatively impact the health systems [12, 13]. Rare disease patients can remain undiagnosed for many years. Their search for a diagnosis can even lead to a diagnostic dead end. In Europe, more than a quarter of patients have to wait more than 5 years obtain a diagnosis (including children) according to a study by EURORDIS [14]. Similar findings were published concerning Spanish [15] and Australian rare disease patients [13]. Counting undiagnosed rare disease patients could be a game-changer as they might be numerous. In French rare disease expert centers, undiagnosed patients can represent up to 50% of the cohort of a given center: these figures are based on an analysis of the French National Rare Disease Registry (BNDMR) [16], which is one of the only registries at EU level to collect data of undiagnosed rare disease patients.

Diagnostic dead ends can also concern patients for whom a diagnostic test is not yet available since the disease has not been characterized and the cause has not yet been identified [17]. To accelerate the research, the International Rare Disease Research Consortium’s (IRDiRC) first goal for 2017–2027 is that “[…] all currently undiagnosable individuals will enter a globally coordinated diagnostic and research pipeline” [18]. Undiagnosed patients can be included in research programs such as SOLVE-RD [19], or one of the Undiagnosed Diseases Network International (UDNI) [20] research programs that aim to reduce the diagnostic odyssey [21, 22]. Such programs are especially focused on finding the genetic etiology of the disease. They involve a combination of deep phenotyping of the patients by interdisciplinary expert panels, exhaustive genetic analysis by utilizing phenotype-driven next-generation sequencing and clinical and genomic data sharing. However, such data are not linked to the hospitals’ electronic records as they are specific to the research program, thus are not part of national statistics.

The European Commission’s European Platform on Rare Disease Registration (EU RD Platform) produced a "Set of common data elements for Rare Diseases Registration" [23] inspired by the French rare disease minimum data set implemented in the BNDMR [24]. Its 16 data elements should be registered by each rare disease registry across Europe, to allow further research and interoperability of rare disease registries. This set of common data elements includes the possibility to describe undiagnosed rare disease patients with their phenotype and genotype. Nevertheless, this possibility does not allow the unambiguous identification of undiagnosed rare disease patients. A common way to formally identify them had yet to be proposed.

In 2017, the European Commission Steering Group on Health Promotion, Disease Prevention and Management of Non-Communicable Diseases selected the codification of rare diseases using ORPHAcodes as a priority area to be implemented as best practice [25], and thus a 'rare disease codification' call was included into the following annual health programme. In 2019, the RD-CODE project [26], supported by a grant in the framework of the Third EU Health Programme and coordinated by INSERM (US14—Orphanet), was launched to support four Member States (Czech Republic, Malta, Romania and Spain) to improve the gathering of information on rare diseases by implementing ORPHAcodes (rare diseases specific nomenclature) into routine code systems. The ultimate goal was to enable a standardized and consistent level of information on rare disease patients to be shared at European level, thus making all rare disease patients, including the ones with no precise diagnosis, visible in health statistics. The RD-CODE work-package 5 (WP5) [27] was in charge of defining rules and guidelines for rare diseases codification using ORPHAcodes across Member States, including tackling the issue of coding undiagnosed patients.

In this paper, we present the RD-CODE project recommendations that are issued from the experience shared by a multi-stakeholder panel of experts for the coding of rare disease patients in health information systems when the specific disease is still unknown, in order to make it possible to count how many patients have an undiagnosed rare disease in a given country/region, produce accurate epidemiological data, and have the possibility to take action to positively impact their diagnostic journey.

## Methods

**Fig. 1:**
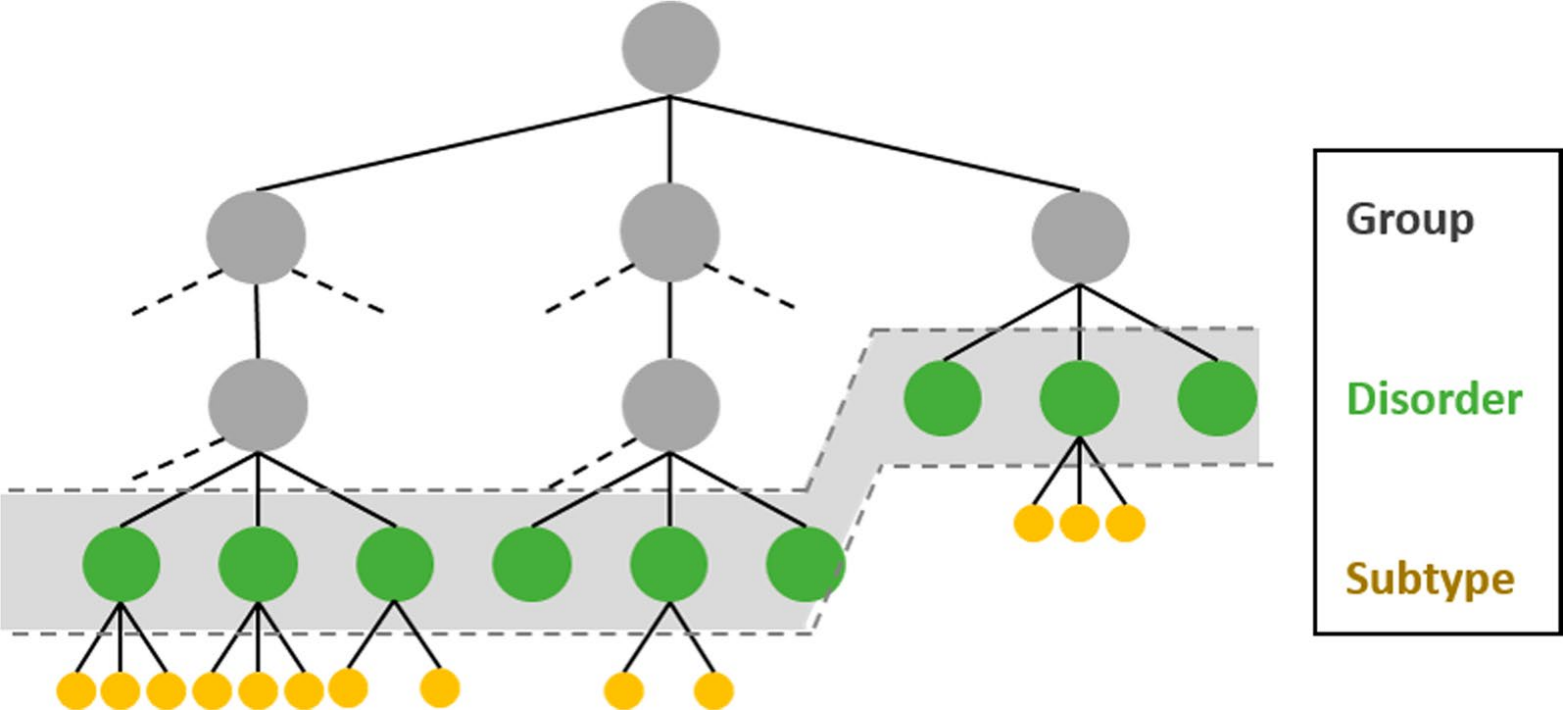
The Orphanet classification representation: groups of disorders, disorders and subtypes

The RD-CODE WP5 members first gathered existing experiences of coding undiagnosed or suspected rare disease patients in health information systems. A review of available literature was initially conducted and summarized in a report. Then all RD-CODE partners, representing different countries (mainly France, Italy, Germany, Spain, Malta, Czech Republic, and Romania, but also Austria, Norway, Belgium, the Netherlands and Portugal), were consulted through several rounds of reviews and two dedicated workshops. This work concluded that the experience in coding undiagnosed patients in electronic health records (EHRs) or patient registries was scarce, mostly research oriented, and prevented possible epidemiological data comparison [28]. The French experience was the only one that dealt with the subject in details. In addition, the definitions of an undiagnosed patient varied from one project to another, and one medical specialty to another. Two additional workshops and experience-sharing sessions with RD-CODE partners were organized in order to ensure a multi-stakeholder perspective. The experts were clinicians-researchers from rare disease expert centers of participating and collaborating countries, members of European Reference Networks (ERNs), patient organizations, and SOLVE-RD project representatives. Ideas from those workshops were structured into definitions and draft recommendations that were sent for validation to the experts. Feedback was integrated and sent back to all the experts for further comments. After several rounds, the final recommendations were produced. Those final recommendations were devised to be consistent with previously published recommendations regarding coding of rare diseases, such as RD-ACTION project recommendations [29] or the EU RD Platform Set of common data elements (CDE) for Rare Diseases Registration. The recommended coding option had to be easy to use and implement, enabling wide implementation through European countries. A way to identify undiagnosed patients without adding any new field in data collection tools was needed. In the framework of the RD-CODE project, the best solution was to rely on the Orphanet classification that was implemented in the participating countries. The entities of the Orphanet classification system (and their unique identifiers) are organized into groups, disorders and subtypes (Fig. 1). A disorder in the database can be a disease, a malformation syndrome, a clinical syndrome, a morphological or a biological anomaly or a particular clinical situation (in the course of a disorder) [30]. A ‘group of disorders’ is not considered as a precise diagnosis because it includes several heterogeneous disorders. Besides, when generating data sets for international comparability, the subtypes can then be aggregated to the level of disorder to provide comparable data.

## Results

### Definitions

The scope of undiagnosed rare diseases first needed to be defined to set a code system for undiagnosed patients and produce recommendations. It was a prerequisite to make transnational statistics possible, based on the same indicators.

In the framework of the RD-CODE project, it was agreed to define “diagnosis” as a process that leads to assigning a disease name to a patient’s clinical situation, or to the undiagnosed status. Thus, the diagnostic suspicion can evolve over time and therefore the possible name attributed to the patient's condition. The closer to the diagnosis confirmation, the more precise it can get, and the granularity level of the ORPHAcode used to describe a rare disease diagnosis can evolve along the patient’s diagnostic pathway. However, in many cases the diagnosis cannot be achieved.

**Fig. 2:**
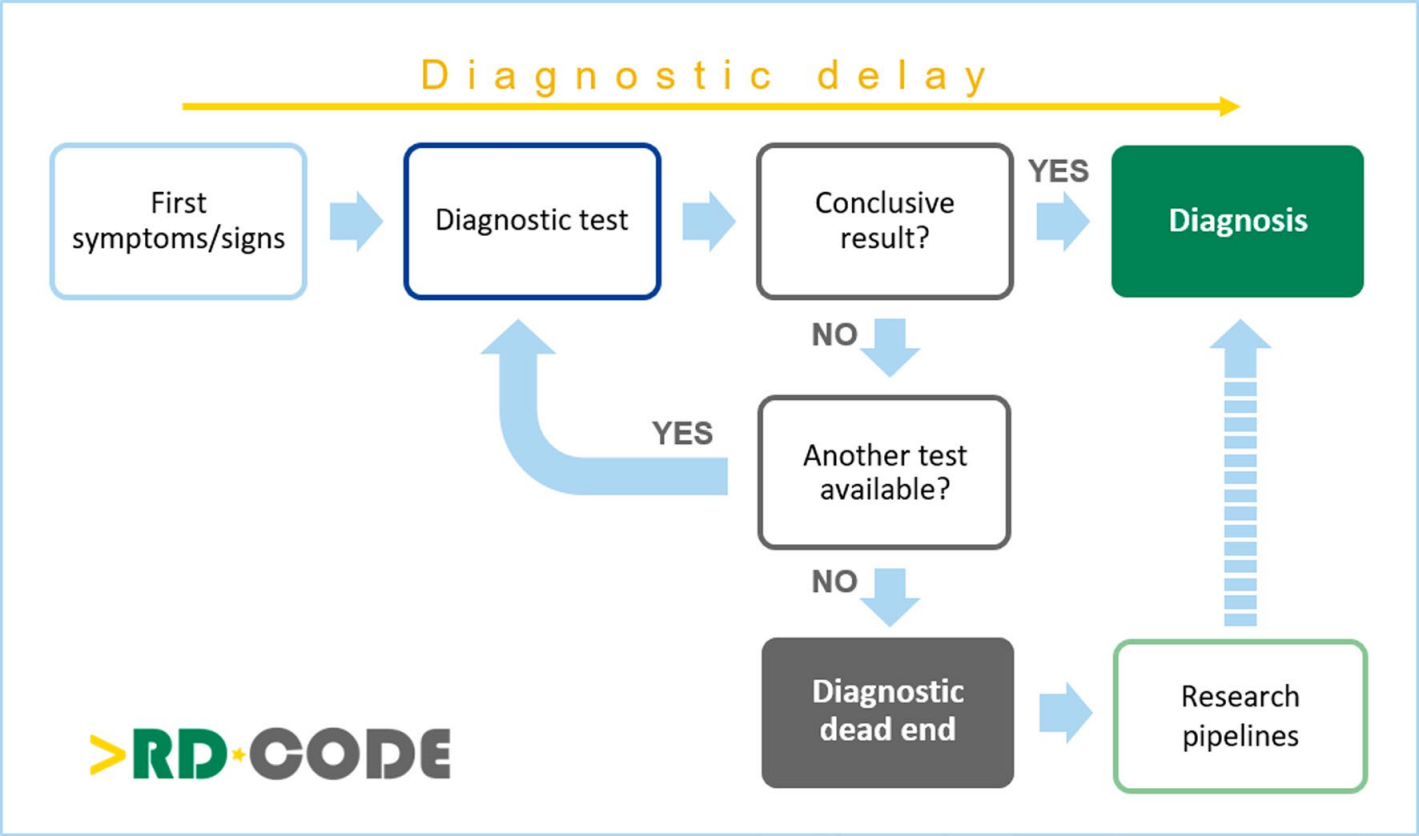
A graphic representation of the workflow toward diagnosis

The diagnostic delay refers to the time during which the patient has not yet been diagnosed. The diagnostic dead end refers to the situation where the patient’s diagnosis was not identified after undergoing all available investigation, according to the state-of-the-art and the diagnostic possibilities in a given country (Fig. 2).

The diagnostic process can include different levels of confirmation: a clinical diagnosis is based on signs and symptoms, whereas an etiological diagnosis is the determination of what causes the disease (genetic defect, environmental impact…). In the framework of RD-CODE, the “undiagnosed patients” term was used to describe patients with no established clinical diagnosis, when “diagnosed patients” have an established clinical diagnosis related to a confirmed rare disease, even without an etiological diagnosis (Table 1). Undiagnosed patients can either have a suspected clinical diagnosis (described with a provisional code) or unknown clinical diagnosis (the disease cannot be named). Patients with a suspected rare disease can usually be identified in health information systems: even without an etiological explanation, it is still possible to describe and code their disease with pending confirmation (also called “working diagnosis”). In contrast, unknown clinical diagnoses (when the disease cannot be identified from patients’ signs and symptoms) cannot be coded because they cannot be named. Phenotype or genotype descriptors could be used to describe the patients’ disease, and consequently be able to browse the system to find them later. However, phenotypic and/or genotyping descriptions are not sufficient to recognize undiagnosed patients in information systems.

**Table 1:** RD-CODE diagnosed and undiagnosed status by type of diagnostic ascertainment. In the framework of RD-CODE, the “undiagnosed patients” term is used to describe patients with no established clinical diagnosis. “Diagnosed patients” have an established clinical diagnosis related to a confirmed rare disease, even without an etiological diagnosis

Clinical diagnosis	Etiological diagnosis	Diagnostic status for RD-CODE
Established	Established	Diagnosed
Established	Unknown	Diagnosed
Suspected	Established	*Undiagnosed*
Suspected	Unknown	*Undiagnosed*
Unknown	Established	*Undiagnosed*
Unknown	Unknown	*Undiagnosed*

How quickly the patient will be diagnosed depends on the condition but also on the expertise and tests available. This is why the up-to-date status of rare disease diagnosis has to be determined by expert centers in the field, to make sure state-of-the-art medical efforts have been made. All undiagnosed patients should not be categorized as undiagnosed rare disease patients, because a rare presentation of a common disorder can be difficult to rule out and requires a confirmed expertise that is scarce. This is why the RD-CODE expert group highlighted the major role of rare disease expert centers, and, in Europe, European Reference Networks, to share experience and knowledge.

In the end, in the framework of the RD-CODE project, we defined undiagnosed patients as patients for whom no clinically known disorder could be confirmed by a rare disease expert center after all reasonable efforts to obtain a diagnosis according to the state of the art and diagnostic capabilities available.

### Recommendations

Three recommendations emerged from the RD-CODE project.

#### First recommendation: diagnostic ascertainment

The first recommendation is that whenever possible, the diagnostic ascertainment should be captured for all rare disease cases. Options such as “Suspected rare disease”, “Confirmed rare disease” and “Undetermined diagnosis” should be displayed. The “Undetermined diagnosis” should only be used when all reasonable efforts to obtain a diagnosis according to the state-of-the-art and diagnostic capabilities available were carried out.

However, this first recommendation might be difficult to implement in existing hospital electronic health records or registries because modifying forms to collect new items might be challenging. The working group still considered valid to code uncertain diagnosis (“working diagnosis”) if the information system allows it, using a generic medical terminology (i.e., HPO, ICD-10, ICD-11, SNOMED CT) or groups of disorders (i.e., Orphanet nomenclature) such as “rare epilepsy”, “rare intellectual disability” or “neurodevelopmental disorder” [31].

#### Second recommendation: new ORPHAcode dedicated to undiagnosed cases

To avoid the burden of adding new fields in data collection tools where ORPHAcoding is implemented, the RD-CODE second recommendation relied on the Orphanet classification. A dedicated ORPHAcode, specifying the “undiagnosed” status, was created to be used alone or in addition to the first recommendation. This new code in the Orphanet nomenclature should allow a specific and unambiguous designation of undiagnosed patients according to the agreed definition: only patients whose diagnosis is coded with the new dedicated ORPHAcode will be considered and counted as undiagnosed.

**Fig. 3:**
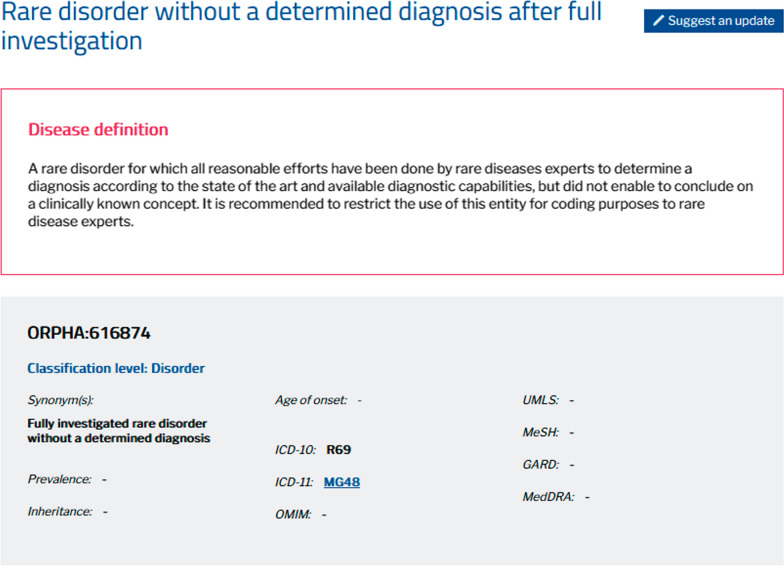
The Orphanet webpage for the new ORPHAcode 616874: Rare disorder without a determined diagnosis after full investigation

The RD-CODE experts established the specifications of this new ORPHAcode and it was created in the Orphanet nomenclature release (Nomenclature Pack) of July 2022: ORPHA:616874 “Rare disorder without a determined diagnosis after full investigation” (synonym: “Fully investigated rare disorder without a determined diagnosis”) (Fig. 3). This code defines a rare disorder for which all reasonable efforts have been done by rare diseases experts to determine a diagnosis according to the state of the art and available diagnostic capabilities, but did not enable to identify a disease. In the classification, it is a disorder (clinical entity) attached to a new dedicated classification head so that it can be easily available in any code system using the Orphanet nomenclature file or the Orphanet Rare Disease Ontology (ORDO) with a simple update. Due to its very particular clinical scope and purpose, this Orphanet nomenclature entity does not carry any scientific annotation (such as genes, natural history, epidemiology, phenotypical descriptions, etc.), except for terminological alignments: ICD-10 R69 “Unknown and unspecified causes of morbidity” and ICD-11 MG48 “Unknown and unspecified causes of morbidity”. However, these ICD-10 alignments are qualified as “narrower-to-broader”: the ORPHAcode is more precise than these ICD codes. This code can be assigned to ad hoc activities in the Orphanet catalogue of expert resources: i.e., undiagnosed research programmes.

To properly use this new code, guidelines were provided and a video [32] was created to help users understand it (see also section *Availability of data and materials*). The code must not be used for known diseases that are not (yet) available in the Orphanet classification. If a code does not yet exist, an online ticketing system (GitHub) that allows new ORPHAcode creation requests to be stored, tracked and shared with others is accessible by registered users [33].

In addition, the code must not be used for coding patients that did not reach yet the end of their diagnostic pathway. This code should only be used after all reasonable efforts to obtain a diagnosis according to the state-of-the-art and diagnostic capabilities available were carried out. Because of the latest point, it is recommended that only experts (in rare disease centers where available) should be allowed to use this code.

#### Third recommendation: additional descriptors in registries

In addition to the recommendations above, registries should also provide a phenotype and a genotype description of undiagnosed patients to be compliant with the EU RD Platform Set of common data elements for Rare Diseases Registration. The third and last recommendation is thus to complete the description of undiagnosed patients in registries with other descriptors. Phenotype description should be carried out using HPO (Human Phenotype Ontology) terms and genotype using HGVS (Human Genome Variation Society) for variants and HGNC (HUGO Gene Nomenclature Committee) for gene names. Additional phenotypic descriptors or broader disease categories could be used (for instance ICD, SNOMED, Orphanet categories…) as well as genetic descriptors (using a system generally recommended to describe chromosomal rearrangements or genomic anomalies).

## Discussion

The RD-CODE project has highlighted the undiagnosed rare disease patients’ diagnostic pathway and the challenge in ensuring that this is properly represented in health information systems. We demonstrated that definitions were needed as the concepts have many layers that were difficult to grasp. Thus, they should be adapted to the final objective, i.e., counting cases, identifying and characterizing undiagnosed patients with rare disease.

One of the challenges of identifying undiagnosed patients as defined here, is the applicability of the solution to Health information systems. The first recommendation alone (adding a qualifier to the diagnostic assertion) requires the creation of a data field in coding software, which could prove undoable in many systems. Furthermore, the qualifiers could be interpreted differently, resulting in unreliable data for statistical reporting and evidence-based action. Ideally, the combined use of the first and second recommendations should be privileged in practice.

Three coding options to identify undiagnosed patients and based on the Orphanet classification were initially considered. The first option to code undiagnosed patients was to use the Orphanet classification group levels (the higher up, the lower the precision of diagnosis definition); the second one was to use one or several new dedicated ORPHAcode(s) specifying the “undiagnosed” status (the one that was finally recommended); and the third one was to use a normalized prefix / marker together with ORPHAcodes. The multi-stakeholder panel of experts’ decision was based on the possibility of each option to reach the first objective of the project: being able to identify undiagnosed patients in electronic health records, to produce comparable statistics in the different countries and take action on them.

The use of the Orphanet classification group levels meant that any patient coded with a group of disorders would have been considered as undiagnosed. However, some patients with identified diseases could, at some point in their diagnostic pathway, have been coded with a group level ORPHAcode, especially when the diagnosis pathway is still ongoing. Therefore, the risk of misuse of the group levels was considered too high and as it could negatively impact the counting of patients, this option was dismissed.

The use of a normalized prefix / marker implied that patients coded with a to-be-determined prefix or marker attached to any ORPHAcode would be considered as undiagnosed. This option required adding a prefix or marker to the Orphanet nomenclature. It would not be supported by the nomenclature itself, so each health information system would have needed to implement it separately from the ORPHAcode fields. In the end, this would have represented a burden comparable to adding a new field (diagnostic qualifier), as discussed above, so this option was dismissed.

The selected option was the creation of one specific code (ORPHA:616874 “Rare disorder without a determined diagnosis after full investigation”). In this implementation option, only patients coded with this code would be considered as undiagnosed. Furthermore, the Orphanet nomenclature current structure and format can be used without changes: a simple annual release update would give access to the new code. The creation of 35 specific codes, one per Orphanet category (head of classification) such as “undiagnosed neurological disease” was also discussed. Creating several codes would not have increased the coding quality or exploitability. They would have been more difficult to use (especially for multi-systemic diseases), would have created heterogeneous coding and required a good knowledge of the Orphanet classification, thus this option was dismissed. Of course, the one-code option has cons, such as the lack of additional information on the patient’s clinical manifestations. However, identifying the cohort is the first step we decided to focus on and additional recommendations to help obtain a more granular picture of the population will be needed, especially in registries. For instance, a way to distinguish undiagnosed patients lacking clinical diagnosis from those lacking both clinical and etiological diagnosis was not covered by our project. To distinguish these two sub-populations among undiagnosed patients, additional information should be included in data collections, based on other terminologies and nomenclatures, as already recommended by the EU RD Platform in the Set of Common Data Elements for Rare Diseases Registration. An evaluation phase would have provided us with strong arguments to validate this choice. We plan to assess the use of the dedicated ORPHAcode and produce the first statistics ever on undiagnosed patients at European level in the upcoming years. To support the new ORPHAcode adoption, a wide-ranging communication strategy was devised. To on-board the rare disease community (i.e., rare disease networks, expert centers clinicians, researchers, patient organizations, and health authorities), several channels were mobilized such as the Orphanet newsletter [34], a video about implementing the recommendations [32], and this article. An anticipated challenge will be to make sure that the code is used according to its definition. Therefore, we support the development of rare disease coding guidelines in rare disease centers that would include this specific ORPHAcode and associated recommendations. This will be facilitated by the structured rare disease networks at national and European level (ERNs) and the strong involvement of patient organizations in relaying this information. The OD4RD project [35] also works to empower hospitals clinicians, coders, medical informatics departments and hospital managers in their use of ORPHAcodes, and in particular at disseminating the recommendations concerning the use of the code for undiagnosed rare disease patients.

## Conclusions

Identifying rare disease patients within health information systems is a key requirement to accelerate patient recruitment for clinical trials or observational and longitudinal data collections such as registries for research and public health purposes. This is also true for undiagnosed patients, who need to be better identified so as to obtain access to research programs (as per the IRDiRC recommendations) such as genomics platforms throughout Europe. Through the ERNs Clinical Patient Management System (CPMS), they could benefit from the shared knowledge of all the whole rare disease expert community in the medical field of interest, thus accelerating their diagnosis. The recognition of the rare disease status can be necessary for patients, even though they do not have a confirmed diagnosis. It can facilitate reimbursement of care, as well as being socially and psychologically empowering. Better rare disease coding and, consequently, an increased visibility of undiagnosed rare disease patients in health information systems can finally inform health authorities about patients’ care pathways and their use of health services, a necessary step in the care planning process and health economic costs impact evaluation. Being able to assess the population of rare diseases patients, including undiagnosed patients, will enable greater and more adequate political measures including financing for care and research programs throughout Europe. The comparable epidemiological statistics in EU countries will be a powerful tool to highlight the needs of patients, clinicians and researchers in the field. In addition to counting patients, a better understanding of patients’ pathways is highly needed. The implementation of the RD-CODE project recommendations will be a game-changer, acting as a catalyst in reaching those goals, especially in countries using the Orphanet nomenclature. Indeed, thanks to the new dedicated ORPHAcode (ORPHA:616874 “Rare disorder without a determined diagnosis after full investigation”) undiagnosed patients will be unambiguously identified. A multichannel communication strategy aimed towards the whole rare disease community is ongoing to support its broad adoption. As a first fruit of this work, in March 2023, Orphanet was honored by the Spanish Federation for Rare Diseases (FEDER) with an award [36] for the creation of this specific ORPHAcode for undiagnosed patients.

## Data Availability

Recommendations for coding undiagnosed rare disease patients and guidelines to implement the newly created ORPHAcode (ORPHA:616874: Rare disorder without a determined diagnosis after full investigation) are available in Deliverable 5.2, downloadable from the RD-CODE website: http://www.rd-code.eu/wp-content/uploads/2022/02/D5.2_RD-CODE_VF2021_FV.pdf. In addition, a video was created to support the implementation of recommendations, also available on the RD-CODE website: http://www.rd-code.eu/guidelines-for-coding-undiagnosed-patients-video/. The new ORPHAcode ORPHA:616874 is embedded in Orphanet Nomenclature annual releases (Nomenclature Pack): https://www.orphadata.com/orphanet-nomenclature-for-coding/
